# My Second Thoughts About Neck Lifts and the “Boston Tuckster” Stitch: A Video Interview With Dr Sumner Slavin

**DOI:** 10.1093/asjof/ojad105

**Published:** 2023-11-29

**Authors:** Sumner Slavin, Lorne King Rosenfield

Welcome to *ASJ*'s next Second Thoughts on First Thoughts offering. Today, Dr Sumner Slavin is our conductor as he takes us for a ride on his neck lift learning curve! As with all great surgeons, Sumner learned from the best—in this case, Joel Feldman. And then, Dr Slavin had some second thoughts, which navigated him to “better” with his variation of the Feldman “tuckster” stitch. In doing so, he has incrementally improved the neckplasty result, both in terms of aesthetics…and safety (Video).

## Supplementary Material

ojad105_Supplementary_Data

